# Implementation of School Nutrition Policies to Address Noncommunicable Diseases in Uzbekistan and Kyrgyzstan

**DOI:** 10.9745/GHSP-D-23-00442

**Published:** 2024-08-27

**Authors:** Olakunle Alonge, Maysam Homsi, Mahnoor Syeda Rizvi, Regina Malykh, Karin Geffert, Nazokat Kasymova, Nurshaim Tilenbaeva, Lola Isakova, Maria Kushubakova, Dilbar Mavlyanova, Tursun Mamyrbaeva, Marina Duishenkulova, Adriana Pinedo, Olga Andreeva, Kremlin Wickramasinghe

**Affiliations:** a University of Alabama at Birmingham, Birmingham, AL, USA.; b Johns Hopkins Bloomberg School of Public Health, Baltimore, MD, USA.; c World Health Organization Regional Office for Europe, Copenhagen, Denmark.; d World Health Organization Country Office, Tashkent, Uzbekistan.; e World Health Organization Country Office, Bishkek, Kyrgyzstan.; f Research Institute of Sanitation, Hygiene and Occupational Diseases, Ministry of Health of the Republic of Uzbekistan, Tashkent, Uzbekistan.; g Department of Disease Prevention and State Epidemiological Surveillance, Ministry of Health of Kyrgyzstan, Bishkek, Kyrgyzstan.; h Tashkent Pediatric Medical Institute, Tashkent, Uzbekistan.; i Kyrgyz State Medical Academy, Bishkek, Kyrgyzstan.; j Republican Center of Health Promotion and Mass Communication under Ministry of Health, Bishkek, Kyrgyzstan.

## Abstract

Noncommunicable diseases (NCDs), including cardiovascular diseases, cancer, and diabetes, account for over 80% of mortality in Uzbekistan and Kyrgyzstan in 2019, and unhealthy dietary behaviors are a major risk factor for NCDs in both countries. In 2021, national stakeholders, in consultation with the World Health Organization, identified school nutrition policies (SNPs) as a major approach to reducing the burden of NCDs in both countries. The SNPs included interventions implemented through a multistakeholder and multisectoral arrangement that aimed to improve the health and nutrition status of children and young people by providing healthy food/beverages and restricting unhealthy foods or beverages in schools. We used a multimethod approach of document review, participatory workshops, and key informant interviews to generate theories of change for the large-scale implementation of SNPs and describe the implementation processes to date, including key implementation and health system challenges, salient implementation strategies, and implementation outcomes in both countries. Multiple pathways for enacting and implementing SNPs successfully were identified. However, significant health system challenges, such as the lack of accountability for contracting and tender processes and coordination among different sectors, continue to hamper the large-scale implementation of these policies in both countries. The pathways, theories, and implementation outcomes identified will facilitate the development of implementation strategies and systematic learning and evaluation around SNPs for NCD prevention and control programs in the Central Asian region and other low- and middle-income countries more broadly.

## Abstract (precis)

School nutrition policies are a promising approach to address the risk factors of noncommunicable diseases, but their large-scale implementation requires clear guidelines for coordination among various actors.

## Key Messages

- School nutrition policies (SNPs) are a major approach to addressing the growing burden of noncommunicable diseases. However, pathways for their large-scale implementation in low- and middle-income countries are not clear.
- In Uzbekistan and Kyrgyzstan, the large-scale implementation of SNPs involves multiple activities, including contracting provision of school meals at specific composition and caloric standards, direct provision of meals through collaboration between public schools and communities, and coordination among various agencies and actors, including the health and education ministries.
- The absence of a consistent coordination mechanism among various agencies and actors, lack of an effective food contracting process and a standard approach for food sourcing to ensure stipulated nutrient standards may hamper implementation of SNPs.

## BACKGROUND

Noncommunicable diseases (NCDs) are the most prominent causes of death worldwide.^1^ They account for 74% of all deaths globally, and 77% of these deaths occur in low- and middle-income countries.^2^ In Uzbekistan and Kyrgyzstan, NCDs, including cardiovascular diseases, cancers, and diabetes, accounted for over 80% of all mortality in 2019,^3^^,^^4^ and an average Uzbek or Kyrgyz citizen has more than 22% chance of dying before age 70 years from NCDs.^5^^,^^6^

Behavioral risk factors, including alcohol consumption, tobacco use, physical inactivity, and unhealthy diet, are the main attributable causes of NCDs.^7^ Of these causes, unhealthy dietary behaviors, including excessive salt intake and high intake of polyunsaturated fats, are ranked as the number 1 and 2 drivers of deaths in Kyrgyzstan and Uzbekistan, respectively.^8^^,^^9^ In both countries, adults aged 20 years and older consumed an equivalent of 14.1g of salt per day (5.63 g of sodium)—2.5 times higher than the WHO-recommended limits of 5 g of salt per day (less than 2 g of sodium/day).^6^^,^^10^

Childhood exposure to NCD risk factors, including the adoption of unhealthy food behaviors and consumption of unhealthy diets that predispose to childhood obesity, leads to NCDs among children and the risk of developing NCDs later in life.^11^^,^^12^ Children adopt unhealthy dietary behaviors (e.g., eating ultra-processed and calorie-dense snacks in between meals, consuming packaged sweetened drinks, and frequently eating highly processed burgers and pizzas with high levels of salts and saturated fats) earlier on.^13^^,^^14^ These behaviors among school-aged children are associated with going to bed after 11:00 pm, prolonged gaming and watching TV during weekdays, skipping breakfast regularly, emotional and psychological distress (e.g., stresses due to academic, relationship, and social media pressures) and may be more common among school-aged children from middle to upper socioeconomic status.^15^^–^^21^

Most children spend 6 hours or more in school and consume as much as half of their daily caloric intake at school.^22^ Moreover, schools provide constant access to children for health promotion initiatives.^23^ Hence, targeting school-aged children with healthy foods, programs to improve healthy dietary behaviors, and improving healthy food options in schools have been recommended as important evidence-based approaches for addressing NCD risk factors, such as obesity.^22^^–^^24^

In this article, healthy foods include recognizable naturally-occurring food ingredients (e.g., fruit, vegetables, legumes, whole grains, fish, lean meat, chicken, eggs, low-fat dairy) combined to provide 60–65 kcal/kg/day for children aged 6–8 years and 35-45 kcal/kg/day for children aged 9 years and older, such that the ratio of proteins, fats, and carbohydrates is set at 1:1:4, or 10–20%, 25%–30%, and 50–55% of the recommended caloric intake, respectively,^25^ of which less than 10% of the carbohydrates is from free sugars, less than 10% of fats is saturated fats (e.g., fats found in fatty meat, palm oil, cheese), and less than 1% of the fats is trans fats (e.g., fats from baked and prepackaged snacks such as cookies and frozen pizza).^25^^,^^26^ Further, healthy food provides less than 5 g of salt/day.

Recognizing the huge burden of NCDs and related dietary risk factors in the Central Asian region, in 2021, the WHO European Office for the Prevention and Control of NCDs (WHO NCD Office), in consultation with national stakeholders in both countries, identified building capacity and providing technical support for the implementation of nutrition policies in schools as a priority for NCD prevention and control. The prioritization exercise was led by the Ministry of Health in both countries with support from the WHO NCD Office and was in line with the WHO Global Action Plan for the Prevention and Control of Non-communicable Diseases 2013–2020.^27^

School nutrition policies (SNPs) targeting children ages 6–11 years and adolescents ages 12–19 years are developed and implemented at the state and local levels in different settings to improve the health and nutrition status of school children and youth.^22^ These policies may include a variety of interventions, such as the provision of nutritional (food-, energy- and/or nutrient-based) standards for menu composition,^11^^–^^13^ direct provision of healthy foods/beverages,^28^^–^^30^ meal plans,^28^ and regulating (restrictions/prohibitions of) competitive foods.^28^^–^^32^ Other priority interventions in SNPs include nutrition health education; screening and monitoring children’s health indicators; staff, administrative, and student training; and other support strategies.^28^^–^^32^

SNPs have been shown to effectively address their targeted behaviors and health outcomes (e.g., reduced mean weight, increased consumption of healthy food, and reduced total fat and sodium intake^11^^,^^33^) in other countries. However, it is yet to be seen if the adoption of SNPs will have similar results in Uzbekistan and Kyrgyzstan. The effective implementation of these policies is also needed to achieve population-level impact in both countries,^34^ but there are gaps in our understanding of the implementation pathways and theories for supporting the effective implementation of SNPs in both countries and most other Central Asian countries.^34^^,^^35^

SNPs have been shown to effectively address children’s and adolescents’ targeted behaviors and health outcomes, such as reduced mean weight and increased consumption of healthy food.

There are currently no comprehensive SNPs being implemented across other Central Asian countries (e.g., Kazakhstan, Tajikistan, and Turkmenistan).^36^ These countries face complex malnutrition challenges among school-aged children, similar to Uzbekistan and Kyrgyzstan, with undernourishment and micronutrient deficiencies persisting in some areas alongside a rise in childhood obesity in others.^36^ Based on meetings and policy dialogues held across the region by the WHO Regional Office for Europe in 2021, only Kazakhstan and Tajikistan have a program that provides free school meals to primary school children, and this only covers a small percentage of schools nationally in Tajikistan. Similarly, only the 2 countries have laws on food safety, as well as regulations on nutrition in schools. It is not clear if the composition of school meals under these programs is evidence-based and if the programs are effective, as well as how to implement these programs at scale.

We describe a multicountry and multistakeholder collaborative process for developing SNPs NCD prevention and control in Uzbekistan and Kyrgyzstan targeting school-aged children ages 6–11 years, identify pathways for the effective implementation of these policies at scale in both countries, and provide lessons for other countries in the Central Asian region. The school children aged 6–11 years were prioritized based on political priorities in Uzbekistan and Kyrgyzstan and are universally targeted irrespective of sex, socioeconomic, disability, or vulnerability status.

## METHODS

**FIGURE 1:**
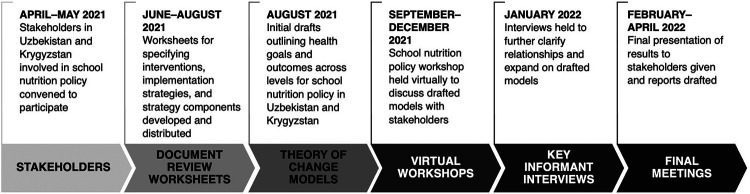
Timeline of Workshop Meetings and Model Development for Implementation of School Nutrition Policies in Uzbekistan and Kyrgyzstan

A multimethod approach combining document review, participatory theory of change workshops, and key informant interviews was applied to fully describe the SNPs, generate theories of change (TOCs) for the large-scale implementation of these policies in both Uzbekistan and Kyrgyzstan, and identify lessons for the effective implementation of these policies in both countries and across the region (Figure 1). The approach used for developing the TOCs that we briefly describe in this article follows a similar approach used for developing TOCs for large-scale implementation of school-based mental health programs.^37^

Between April 2021 and April 2022, relevant stakeholders involved with SNPs in each country were identified and convened to iteratively describe key interventions that were adopted as part of the SNPs, how and why these interventions were incorporated, and make explicit the pathways by which these interventions were expected to lead to their anticipated outcomes in both countries. This effort was led by the WHO NCD Office and facilitated by WHO Country Offices, which have been working to support the planning and implementation of SNPs across various sectors in both countries. Stakeholders identified in Uzbekistan (N=16) and Kyrgyzstan (N=21, n=7 in a core group and n=14 in an expanded group) included representatives from the following agencies: Ministry of Health, Ministry of Education, Ministry of School and Preschool Education, WHO Country Office, schools, teachers, school directors, and professional associations (e.g., family physicians) (Tables 1 and 2). The interest of parents was expressed by the teachers and school directors.

**TABLE 1.** Stakeholders Convened for School Nutrition Policy Implementation in Uzbekistan

**Agency**	**Role/Position and Level**
Ministry of Health	
Republican Specialized Scientific and Practical Centre of Paediatrics	Chief pediatrician, Director, and Head of National Professional Association of Paediatricians
Centre for Professional Development of Medical Workers/Ministry of Health	Chief specialist for Children in Education Institutions/ and Head of the Department of Paediatrics and Nutrition of Children
Tashkent Paediatric Medical Institute	Teacher
	Researcher
	Associate Professor
Republican Specialized Scientific and Practical Centre of Paediatrics	Researcher
	Pediatrician
Public fund for the support of children State Health Insurance Fund under the Cabinet of Ministries	Head of the Department, Senior Specialist
Ministry of Preschool and School Education, Department for Coordination of Activities of General Secondary Education Institutions	
	Department Head
	Deputy Head
	Senior Specialist
	Senior Specialist
Private School “Lieder”, Tashkent city	Teacher
School #5, Fergana city, Fergana region	Director
School #25, Karshi city, Kashkadarya region	Director
World Health Organization Country Office	Technical Officer, National Professional Officer on Noncommunicable diseases

**TABLE 2.** Stakeholders Convened for School Nutrition Policy Implementation in Kyrgyzstan

**Agency**	**Role/Position and Level**
**Core group**	
Ministry of Health	Specialist of the Public Health Department
Ministry of Education and Science	Senior specialist, lead on School Meals Programme
Department of Disease Prevention and State Epidemiological Surveillance	Specialist on child and adolescent hygiene
Republican Centre of Health Promotion and Mass Communication	Nutrition specialist
United Nations World Food Programme	Programme Policy Officer of School Meals Programme
Chui boarding school for orphans and children left without parental care	Director
World Health Organization Country Office	Technical Officer, National Professional Officer on Sexual, Reproductive, Maternal, Newborn, Child, and Adolescent Health
**Extended group**	
Ministry of Education and Science	Senior specialist
Ministry of Health	Chief affiliated pediatrician of the Ministry of Health
Department of Disease Prevention and State Epidemiological Surveillance	Head of department for supervision of food safety and prevention of alimentary diseases
Republican Center of Health Promotion and Mass Communication under Ministry of Health	Specialist on school health program
Independent expert	Independent expert on school nutrition policy
World Health Organization Country Office	Technical Officer – National Professional Officer on Noncommunicable diseases
United Nations World Food Programme	Nutrition specialist
Association on Adolescent Health	Director
UNICEF	Health and nutrition specialist
Kyrgyz State Medical Academy	Professor, Lead on the Academic Network of the Scaling up Nutrition Platform in Kyrgyzstan
Independent expert	Former consultant on school nutrition policy to Food and Agriculture Organization of the United Nations and the World Food Programme
Food and Agriculture Organization of the United Nations	National project coordinator
Aga Khan Foundation	Health and nutrition program manager
Mercy Corps	Director of programs

A document review worksheet was developed using Proctor’s framework for specifying implementation strategies^38^ and the Consolidated Framework on Implementation Research^39^ to extract information on the interventions included as part of the SNPs in both countries and the process, settings, and activities involved in their implementation (Supplement). Stakeholders convened in the previous step were asked to complete the worksheets drawing on their experience and information from accessible policy documents and data sources based on their role as high-level ministry officials (9 government documents in Uzbekistan and 5 in Kyrgyzstan).

Based on the information extracted from the worksheets, initial drafts of theory of change (TOC) models were developed by the research team for specific interventions that formed the SNPs in Uzbekistan and Kyrgyzstan. The TOC models were developed by listing and sequencing the relevant overall health goals and outcomes at various levels (individual, community, and school) linked to the interventions and the policy advocacy activities that supported the adoption of the interventions. This approach followed the convention for developing TOC described elsewhere.^40^ The TOC models were further developed and validated with the stakeholders via a 90-minute participatory virtual workshop in each country. The workshops were facilitated by a study team member with expertise in implementation research and conducting TOC workshops. The workshop discussions focused on clarifying the specified outcomes and the relationships among the outcomes given the ongoing implementation experience in both countries.

After the workshop, interviews were conducted with 2 key informants to further clarify the salience of specific facilitators and barriers to the implementation of SNP in both countries. The interviews were conducted virtually over Zoom by the study team, and each lasted about 60 minutes.

Last, a combined dissemination meeting was organized with the WHO NCD Office, WHO Country Offices, and key stakeholders from Uzbekistan and Kyrgyzstan as well as other countries from the WHO European Region to review and validate the final drafts of the TOCs, their key assumptions, implementation challenges and strategies, and contextualize the TOCs for the region (i.e., qualitatively evaluate whether the relationships, challenges, and strategies described were relevant for other similar countries). All workshops, meetings, and interviews were conducted in English and Russian with simultaneous interpretation.

We describe the SNP interventions that were implemented in both countries, the TOCs and implementation outcomes for supporting their large-scale implementation, key implementation challenges and strategies, and some key assumptions for successful implementation.

## IMPLEMENTING A SCHOOL NUTRITION POLICY IN UZBEKISTAN

### Priority Interventions

The priority interventions as part of the SNP in Uzbekistan included the provision of healthy food for school-aged children through a school meal program and restrictions/prohibitions on the sale of foods in schools outside of the meal program. These interventions were accompanied by nutrition health education delivered through changes to the school curriculum to discourage unhealthy eating behaviors and facilitate the formation of healthy eating habits among school-aged children.

The priority interventions as part of the SNP in Uzbekistan included providing healthy food for school-aged children through a school meal program and restricting/prohibiting the sale of foods in schools outside of the meal program.

The interventions were developed by the Government of Uzbekistan in consultation with the relevant ministries (health and education), departments, and stakeholders, including parents and school administration. Under the program, the provision of healthy food for each school was outsourced to contractors who prepared and supplied the meals to schools according to stipulated standards. The meals were based on the following food products: meat, milk, butter, vegetable oil, wheat bread, eggs, cheese, quark (a type of curd cheese), vegetables, and other dairy products. Meals were guided by the recommended daily food consumption standards: with the ratio of proteins, fats, and carbohydrates set at 1:1:4 or 10–20%, 25%/–30%, and 50%–55%, respectively, and the ratio of calcium to phosphorus at 1:1.5.^25^ The meals also included a piece of fruit as a side and were prepared to provide a proportion of the daily recommended caloric intake 60–65 kcal/kg for children aged 6–8 years and 35-45 kcal/kg for children aged 9 years and older.^25^

### Implementation Process and Activities

The meals were provided based on an approved monthly meal plan developed by the contractor in accordance with the daily nutritional and hygiene standards set by the Ministry of Health. Monitoring and enforcement measures were done to ensure that the contractors complied with the hygiene standards in terms of the food products and how they were prepared, the equipment that was used, and that the kitchen staff complied with the established rules for working in a food preparation area. The main agency responsible for monitoring and enforcement of standards was the Sanitary-Epidemiological Welfare and Public Health Service under the Ministry of Health (now officially known as the Sanitary-Epidemiological Welfare and Public Health Committee since January 2023). The committee also took part in selecting the food contractors based on pre-specified quality, hygiene, and safety criteria. The government covered the cost of meals for all school-aged children during the school year, as well as during the weekends and holidays for children studying at boarding schools.

At the time of writing, the program was implemented only in schools that were part of the Agency for the Presidential, Creativity and Specialized Schools under the Cabinet of Ministers of the Republic of Uzbekistan, which encompassed 141 specialized day schools and 19 specialized boarding schools in 12 regions of Uzbekistan and the capital city, Tashkent (specialized schools combine both elementary and secondary schools). Children studying at specialized boarding schools received 5 meals a day, and those at specialized day schools received 2 meals a day. Further, in pilot regions of the Republic of Karakalpakstan and Khorezm, the government covered single daily meals for children from grades 1–4 (aged 6–10 years). Karakalpakstan and Khorezm are some of the most remote and least developed regions of Uzbekistan. In all, around 14% of the targeted school-aged children in Uzbekistan participated in the program. There were plans to build another 25 specialized boarding schools by 2024 and introduce the school meals program to all schools to cover school-aged children aged 7–11 years in Uzbekistan.

### Theory of Change Model in Uzbekistan

**FIGURE 2:**
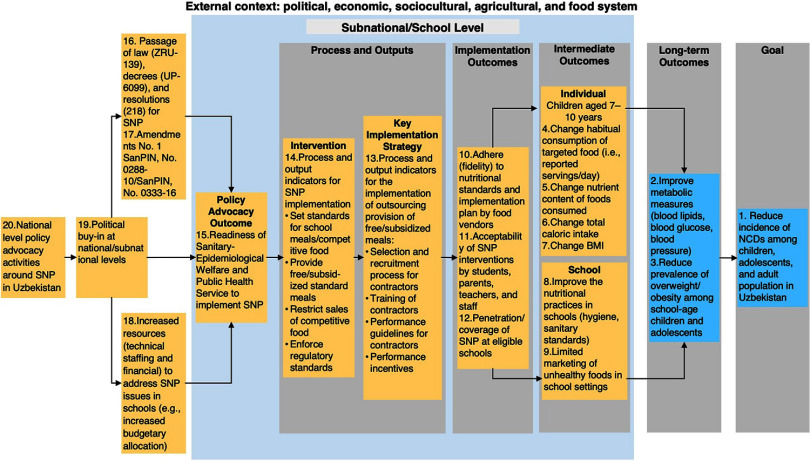
Theory of Change for School Nutrition Policies in Uzbekistan^a^ Abbreviations: MOH, Ministry of Health; NCD, noncommunicable disease; SNP, school of nutrition policy. ^a^ Key assumptions: (1) National MOHs and other stakeholders willing to prioritize SNP, (2) availability and motivation of personnel connected with school environment to implement SNP, (3) Most children attend schools under the Agency for the Development of Presidential, Creative and Specialized Schools System, and (4) others. Yellow boxes are preconditions and numbers indicate stakeholders’ relative importance ranking of preconditions/outcomes needed to achieve the goal, with 1 being the most important.

The long-term goal for implementing the SNP interventions in Uzbekistan was to reduce the incidence of NCDs among children by targeting a reduction in the incidence of overweight and obesity among school-aged children, which had one of the worst prevalences observed (26% among both females and males) among countries categorized as NCD predominant, and reduction in cardiovascular diseases, stroke, diabetes, and cancers associated with poor nutrition later in life in adults. For the individual-level intermediate outcomes, the SNPs were anticipated to improve the habitual consumption of the targeted food at the nutrient composition and caloric standard and lead to changes in body mass index. For school-level intermediate outcomes, the SNPs were anticipated to improve the nutritional practices in schools and restrict access to unhealthy foods in school settings. These intermediate outcomes were mediated by implementation outcomes, including implementation fidelity to the nutritional standards and implementation guidelines by the food contractors, acceptability of the SNP interventions to key stakeholders (students, parents, teachers, and staff), and sufficient coverage of the SNP across eligible schools in Uzbekistan (Figure 2).

The crucial role of the Sanitary-Epidemiological Welfare and Public Health Service under the Ministry of Health as a key implementer (both in managing school meal contracts and executing the policies around food restrictions in school) was clear earlier on, and their readiness (including human resources and technical and financial capacity) to undertake this role was identified as a key policy advocacy outcome along the pathway for achieving the long-term goal of the SNP interventions (Figure 2).

During the TOC development exercise, critical gaps in the implementation pathways for the SNP in Uzbekistan were discovered between the policy advocacy outcome and SNP interventions and between SNP interventions and the implementation outcomes, which may continue to impede the long-term goal as the program is currently being implemented. For example, the capabilities of agents within the Sanitary-Epidemiological Welfare and Public Health Service to manage food contracts while enforcing sale restrictions of competitive foods across schools could not be ascertained. Also, the tendering process for selecting contractors, training them, and monitoring their performance over time was not clear, and this process would likely vary across different subnational units. Hence, for the implementation pathways to work, additional implementation strategies may be necessary, including expanding the capability of the Sanitary-Epidemiological Welfare and Public Health Committee, developing and adopting clear training and performance guidelines for the contractors, provision of performance incentives to ensure that quality standards are met, and developing a monitoring plan to collect and track data on program performance and outcomes.

External context played a major role in how the implementation pathways played out. For example, a favorable sociopolitical context in Uzbekistan paved the way for the provision of policy instruments and the allocation of funds for the healthy school meals program.^41^ Similarly, the health system played an influential role in ensuring that both the intermediate and long-term health outcomes at the individual level were achieved by providing primary and secondary health care services (e.g., nutritional status tracking, nutritional counseling, diagnoses and treatment of nutritional diseases) and delivering nutrition interventions could shape nutritionally related health outcomes with or without the SNP.^42^^,^^43^ The food systems similarly influenced the interventions and implementation strategies, including the sourcing of food products constituted in the meal program; the pricing, marketing, and distribution of these products; and the availability, pricing, marketing, and distribution of unhealthy alternatives.^44^ Moreover, the food system governed the production and access to various types of food outside of schools, including within the home environment, and contributed directly to nutritional practices at different levels.

Key assumptions that underlie the TOC for the large-scale implementation of SNP in Uzbekistan include the willingness of relevant ministries and stakeholders to prioritize SNP, availability of a sufficient number of competent contractors to implement SNP, stable food and commodity prices, and sufficient population coverage of the SNP for school-aged children in Uzbekistan.

## IMPLEMENTING A SCHOOL NUTRITION POLICY IN KYRGYZSTAN

### Priority Interventions

The prioritized SNP in Kyrgyzstan entailed the provision of school meals to students in grades 1–4 (which overlap children aged 6–10 years) at the state and municipal public schools and the restriction of the sale of unhealthy food in schools. These interventions were done in accordance with decree no. 372 of the President of Kyrgyzstan, which provides regulations on the organization of student meals in state and municipal public schools in Kyrgyzstan, and government decree no. 673, which mandates statutory budgetary allocations for the provision of student meals for all students of grades 1–4 in state and municipal public schools in Kyrgyzstan.

About 18% of children in Kyrgyzstan suffer from malnutrition.^45^ The triple burden of malnutrition marked by stunting and vitamin and micronutrient deficiencies has been recognized as a major health concern affecting children in the country and is associated with about 22% of child mortality.^45^ A relatively high percentage of children and adolescents have iron and folate deficiencies (e.g., over 80% of females aged 10–18 years are folate deficient and about 40% of them are vitamin D deficient or insufficient).^46^ Hence, the school meals provided under the program are required per Kyrgyzstan law to be prepared from fortified food sources, such as fortified flour enriched with iron and B vitamins, including folic acid.

### Implementation Process and Activities

In 2016, the Institute of Nutrition in the Russian Federation first developed technological charts that provided guidance on food and menu standards and revised them in 2019. They were approved by the Kyrgyzstan Ministry of Health and Ministry of Education and Science, which provided the charts to state and municipal public schools to develop menu plans following the recipe, nutrient, and caloric standards as described in the charts.

As of the writing of the article, 74% of the state and municipal schools in Kyrgyzstan (about 55% of targeted school-aged children) implemented the optimized school meal program (also referred to as the hot meal program), led by the Ministry of Education and Science in cooperation with the Ministry of Health and food assistance and technical support from development partners (UN World Food Programme also known as WFP and Mercy Corps). The country is administratively divided into regions and districts within regions. The intervention was funded and implemented through the Department of Education (DOE) operating at the district level across the country. The DOE administered relevant contracting tenders and oversaw the contractors that provided free meals according to the menu plans developed by the schools. Contractor selection was done by a DOE committee consisting of physicians, nurses, and representatives from local government and educational authorities. The heads of local state administrations and local self-government bodies organized the purchase of food products in accordance with the legislation on public procurement.

The remaining 26% of the public schools (encompassing about 30% of school-aged children in Kyrgyzstan) received only 7–10 soms (US$0.08–0.11) per child per day directly from the Government that could only cover milk, tea, kompot (fruit drink), and bun or cookies. Although in 2024, the per capita amount was increased to 14 soms (US$0.16), these schools continue to face challenges with the shortage of adequate kitchen equipment and water supply for meal preparation.

### Theory of Change Model in Kyrgyzstan

**FIGURE 3:**
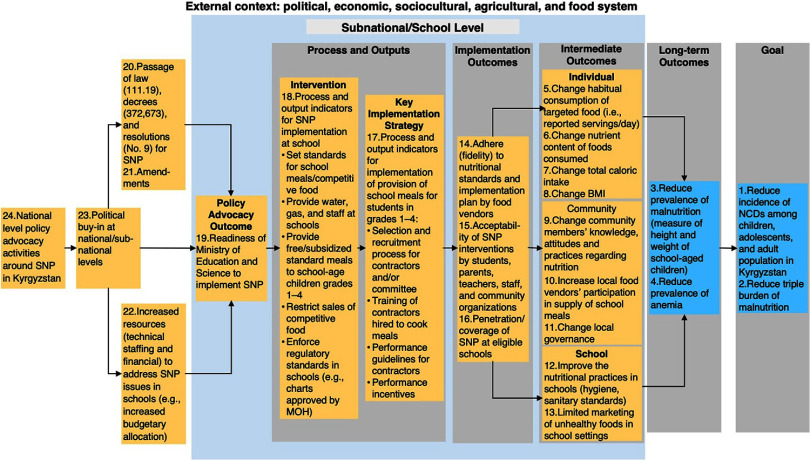
Theory of Change for School Nutrition Policies in Kyrgyzstan^a^ Abbreviations: MOH, Ministry of Health; NCD, noncommunicable disease; SNP, school of nutrition policy. ^a^ Key assumptions: (1) National ministries of health and education and other stakeholders willing to prioritize SNP, (2) availability and motivation of personnel connected with school environment to implement SNP. Yellow boxes are preconditions and numbers indicate stakeholders’ relative importance ranking of preconditions/outcomes needed to achieve the goal, with 1 being the most important.

The stakeholders in Kyrgyzstan prioritized reducing the prevalence of anemia (32% among females and 31% among males), which is one of the worst observed,^47^ in addition to addressing overweight and other forms of malnutrition (Figure 3). The main causes of anemia among children and adolescents are iron and folate deficiencies, occurring mostly among females.^46^ Anemia is regarded as an NCD that directly affects children and adolescents^48^^,^^49^ and is associated with NCDs, such as ischemic stroke, later in life.^50^^–^^52^

The main implementer in Kyrgyzstan was a multiagency group comprising the Ministries of Health, Education and Science, WFP, and Mercy Corp. The healthy meal plan development was also devolved to public schools in Kyrgyzstan. Meals were provided in Kyrgyzstan partly through contracts with food vendors and direct provision by schools with community involvement.

During the TOC exercise, critical gaps in the SNP implementation pathways in Kyrgyzstan centered around the capabilities of the DOE to manage school meal contracts and tendering process, training and developing performance guidelines for contractors and state and municipal public schools, and monitoring and enforcement of quality standards. Additional gaps observed in the SNP pathway included the absence of a consistent coordination mechanism between public schools and communities where meals were directly provided by schools and the lack of a standard approach for food sourcing to ensure stipulated nutrient standards were met. The additional implementation strategies and assumptions previously described for the SNP in Uzbekistan also applied to the SNP in Kyrgyzstan. In addition, standardization of the food sourcing approach across contractors and schools and the establishment of a coordination mechanism between public schools and communities may be needed to ensure the nutritional standards and implementation quality of SNP in Kyrgyzstan.

## DISCUSSION

SNPs were being implemented to address the growing burden of NCDs in Uzbekistan and Kyrgyzstan. SNPs adopt a life course approach by prioritizing healthy nutrition and behaviors for school-aged children to minimize NCD risks for them as youth and adults later in life. For example, the SNP was expected to lead to a reduction in the incidence of NCDs and risks (e.g., obesity and anemia) among school-aged children in the short- to mid-term and adoption of healthy food habits among these age groups, which will contribute to the reduction in the incidence of NCDs associated with nutrition later in life. The SNP interventions did not specifically target known sex and age differences in these outcomes. For example, a higher prevalence of overweight and obesity among school-aged boys compared to school-aged girls and among those aged 5–9 years compared to those aged 10–19 years would likely persist^53^ but would reduce for all school-aged children irrespective of sex and age over time. The interventions were being implemented through multistakeholder partnerships and multisectoral collaboration in both countries to achieve large-scale impact.

SNPs adopt a life course approach by prioritizing healthy nutrition and behaviors for school-aged children to minimize NCD risks for them as youth and adults later in life.

SNPs in both countries share some similarities. For example, both are based on nutritional standards consistent with available evidence (Korea, United States, and 5 continents)^54^^,^^55^ and entail the provision of free healthy meals to school children and sale restriction of competitive foods in schools. Yet, SNPs differed in several ways. Whereas both countries are classified as NCD-predominant countries according to the Lancet Commission on Adolescent Health and Well-Being, the underlying NCD risks among children and adolescents differed slightly between countries.^47^ The political priorities for children and adolescent health in both countries were also different. Hence, the goal of reducing NCD in both countries was the same, but the pathways to achieving this goal (defined by the long-term outcomes) were different. For example, stakeholders in Uzbekistan prioritized reducing the prevalence of overweight/obesity among children and adolescents. Implementation activities had significant differences, which had implications for the implementation pathways to their large-scale effectiveness in both countries (Table 3). First, the main implementing organization was different in both countries, which carries with it differences in organizational culture, climate, and behavior that influenced the implementation process. The scale of the implementation was also different, essentially covering all schools in Kyrgyzstan but only selected schools under a special presidential program or a pilot project in Uzbekistan. This difference in scale, in turn, influenced the program’s implementation arrangements. In Uzbekistan, the program was implemented mainly through a centralized and standardized contracting process, whereas Kyrgyzstan used multiple approaches, including both contracting processes and direct provision of meals by some schools with significant devolution of power and control to schools and local communities. The direct provision of meals by some schools in Kyrgyzstan may have become necessary because of the shortages of potential food vendors in the country to rapidly scale the program. The differences in scale of the program may have also impacted access to food sources to maintain the nutritional standards, with significant challenges in food sourcing in Kyrgyzstan relative to Uzbekistan. Whereas there is no single approach to implementing SNPs and large-scale implementation of programs is often opportunistic in most countries, the different SNP implementation arrangements provide important guidance on likely pitfalls to other countries in the region and emphasize the need for scale-up of nutritional programs to be planned and systematic.

**TABLE 3.** Commonalities and Differences in School Nutrition Policies in Uzbekistan and Kyrgyzstan

	**Commonalities**	**Unique Features in Uzbekistan**	**Unique Features in Kyrgyzstan**
**Key SNP intervention components**			
Provision of meals	Provision of free standard meals.	School-aged children in schools managed by Agency for the Development of Presidential, Creativity and Specialized Schools under Cabinet of Ministers of the Republic of Uzbekistan initiative.	Students in grades 1–4 at the state and municipal schools.
	Restrictions/prohibitions on the sale of competitive foods in schools.		
	Programs developed by the government in consultation with World Health Organization and other key country stakeholders (professional organizations, nongovernmental organizations).		
Main implementer		Sanitary-Epidemiological Welfare and Public Health Committee, an agency of the Ministry of Health.	Department of Education, an agency of the Ministry of Education and Science.
Meal provision		Mainly contracted out.	Contracted out for 74% of public schools and directly provided to 26% public schools.
Contract administration		By agency under the Ministry of Health through a competitive bidding process.	By Department of Education under the Ministry of Education and Science.
Current implementation strategies included as part of SNP	Provision of standard meals outsourced to contractors. Cost of meals covered by government.	Outsourcing of meals entails thorough selection, recruitment, training, monitoring and compensation to contractors through the Sanitary-Epidemiological Welfare and Public Health Committee.	The heads of local state administrations and local self-government bodies organize the purchase of food products in accordance with the legislation on public procurement.
**Key outcomes and mechanisms in Theories of Change**			
	SNP targets reduction of the incidence of noncommunicable diseases among children and adults.		
Long-term outcomes	Reduction in the prevalence of malnutrition among school-aged children at the population-level.	Improvement in metabolic measures.	Improvements in micronutrients and include reduction in the prevalence of anemia, folic acid and vitamin D deficiencies among school-aged children and adolescents.
Intermediate outcomes	Individual level: Changes in habitual consumption of target foods (including the nutrient and caloric standards) School level: improvements in nutritional practices and limited marketing of unhealthy foods in school settings.		Community level: Improvement in local governance i.e., increased allocation of funds for school nutrition in their jurisdiction. Increase in participation of local food vendors in supply of school meals from local communities. Positive change in community’s behavior regarding nutrition and associated practices.
Policy Advocacy outcome		Readiness (human resources, technical, and financial capacity) of the Sanitary-Epidemiological Welfare and Public Health Committee under the Ministry of Health to implement the SNP.	Readiness (human resources, technical, and financial capacity) of the Department of Education under the Ministry of Education and Science to implement the SNP in cooperation with the Ministry of Health and technical support from development partners (World Food Programme and Mercy Corps).
Implementation outcomes	Improved adherence to the nutritional standards and implementation plan by food vendors. Increased acceptance of SNP interventions by all stakeholders. Increase in penetration of SNP at eligible schools.		
**Implementation challenges**	Lack of coordination and concordance among key players in establishing standards for school meals/nutrition. Lack of performance and training standards for contractors, schools, and local communities involved in SNP. Issues in enforcement of nutritional, implementation, and regulatory standards. Limited system for tracking and monitoring performance of contractors.	Limited political buy-in at the national/sub-level levels. Limited resources available for expansion of program to schools other than ones that are part of the Agency for the Development of Presidential, Creative and Specialized Schools initiative.	Limited pool of contractors, and lack of consistency in tendering process for hiring contractors. Lack of a consistent procedure for developing meal plans across schools and vendors. Variable food sourcing approach across contractors and state and municipal public schools which gives rise to variable quality of the meals being provided. Lack of a coordination mechanism between state and municipal public schools and communities in schools where meals are being directly provided.
**Key Theory of Change assumptions**	Willingness of national ministries of health and education and other stakeholders to prioritize SNP. Availability and motivation of competent contractors to implement SNP. Stable food and commodity prices.	Only around 10% of children in Uzbekistan attend schools under the Agency for the Development of Presidential, Creative and Specialized Schools.	Sufficient population coverage of SNP for school-aged children in Kyrgyzstan.

The different SNP implementation arrangements provide important guidance on likely pitfalls to other countries in the region and emphasize the need for scale-up of nutritional programs to be planned and systematic.

In Kyrgyzstan, there are debates around whether to contract or not to contract for school meals provision, especially given concerns around the fragmentation in contracting arrangements, inefficiencies in tender systems, lack of oversight for contractors and food standards, corrupt contracting practices, and limited human resources capacity. While contracting allows for rapid scale-up of the program compared to the direct provision of meals by schools with community involvement, the latter facilitates parents’ and communities’ engagement and ownership of the program and may be associated with better quality of food. Indeed, both strategic approaches have their advantages and disadvantages. However, if the contracting will be retained in the future, it will be important to develop a system for defining and enforcing contracting arrangements and tender systems to achieve impact.

Currently, contractors simply bid to supply meals based on a monthly nutrition plan and guidelines (which specify the nutrient and calorie standards) developed by the main implementer based at the Ministry of Health in Uzbekistan and Ministry of Education in Kyrgyzstan. The bid application is reviewed by a committee based on cost, quality, and safety standards, and the contractor is responsible for sourcing the food items from the market. However, performance and quality standards of the supplied meals are not regularly monitored and enforced by the committees, which is one of the main challenges facing the program in both countries.

If the contracting challenges cannot be addressed in the short- to mid-term, efforts targeted at addressing the shortcomings of the direct food provision may be prioritized, including establishing independent stores/warehouses that could be certified to provide fresh foods to schools to address the food sourcing challenges and setting up clear standards and protocols for school and community engagement to facilitate direct meal provision. For example, the certification of independent stores/warehouses could involve third-party food safety audits that verify that fresh fruits and vegetables are produced, packed, handled, and stored in ways that minimize risks of microbial contamination based on regulatory practices drawn from experiences of other countries (e.g., U.S. Department of Agriculture, European Union General Food Law and Regulation, United Kingdom’s Safety Act). These regulatory practices have been shown to be effective in improving the knowledge, attitudes, and behaviors of food handlers and distributors.^56^

In both countries, the SNP implementation pathways operated at different socioecological levels through various intermediate outcomes, including improvement of nutritional practices in schools and sale restriction of competitive foods at the school level; changes in nutritional knowledge and practices, increased participation of local community members in meal production and local governance of schools at the community level; and changes in consumption of targeted foods, metabolic measures, body mass index, and prevalence of malnutrition at the individual level. These intermediate outcomes in both countries are similarly mediated by implementation outcomes, including the acceptability of the SNP interventions by stakeholders (students, parents, teachers, school administrators, and community members); adherence to the nutritional standards and implementation plan by food vendors and schools; and spread of the SNP program among eligible schools. In both countries, some of the implementation strategies and support systems needed to facilitate these outcomes are not currently in place, such as training contractors; providing reference standards, performance guidelines, and incentives to guide the contracting process and improve quality; and developing information systems to collect data and assess changes in the outcomes over time.

The challenges with implementing SNPs in Uzbekistan and Kyrgyzstan extend to other countries in the Central Asian region. For example, at a regional policy dialogue on SNP organized by the WHO Regional Office for Europe in 2021, many participants from different Central Asian countries highlighted the weak coordination between the Ministry of Health and the Ministry of Education. Similarly, the lack of performance standards for contractors, schools, and communities involved in SNP and lack of adoption or enforcement of nutritional standards continue to plague the success of SNP in Kazakhstan, one of the only countries from the region that has a school meal program.^57^ Hence, the lessons and strategies discussed in this article for SNP in Kyrgyzstan and Uzbekistan may have relevance to other countries in the region.

The availability and cost of different types of food in schools are influenced by the food system,^58^ including actors involved in agricultural production, food importation, and aid capacities. For example, the Global Food Security Index ranked Uzbekistan 73 of 113 countries in 2022 and lists some of the biggest challenges to food availability, including agriculture producer prices, empowering of women farmers, and limited irrigation infrastructure.^59^^,^^60^ These issues may have been further exacerbated by the armed conflict in the region and inflation, which has pushed up food and agriculture input prices globally.^61^ While the current average cost of providing school meals in the region is unknown (estimated at US$0.10–0.15 in Kyrgyzstan in 2017),^62^ SNP will clearly require considerable and continuous funding to be sustainable. Hence, innovative funding strategies may be needed to sustain the school meals program under SNPs in both countries and across the Central Asian region.

Further, the production and distribution of food are governed by market forces and sensitive to market fluctuations in both countries and across the region. The capacity to process food in both countries is also limited despite the agricultural sector being a leading employer.^63^^,^^64^ Agents from the education and health sectors interact with food suppliers through market interactions, and none of the countries in the region, except for Kyrgyzstan, has any regulations on how unhealthy food (e.g., highly processed food rich in trans-fat) are marketed to children outside of schools.^36^ Hence, broader policies, including those ensuring fair market prices, consumer protection, and safe marketing practices, are needed to ensure that the goals of the SNP are achieved.

While still likely to evolve, the TOCs, outcomes, strategies, and pathways we describe are relevant for initially guiding a systematic evaluation and learning on large-scale implementation of SNP in both countries and other low- and middle-income countries. For next steps, the description of the SNP and implementation processes will be used to package and test selected implementation strategies for addressing gaps in the TOCs and to develop tools and mechanisms to systematically learn and improve the policy outcomes in both countries and guide the adoption of similar policies in the region.

### Limitations

The approach as applied in this article has some limitations. For example, the TOC workshops were conducted virtually (due to COVID-19 pandemic restrictions), which may have limited the discussions around the programs and their implementation pathways and the ability to form consensus on key outcomes. We tried to overcome this limitation by prepopulating the TOC diagrams based on the document synthesis before the meetings so that the workshop time could focus on validating the nature and forms of different outcomes and relationships among them. It should also be noted that the TOC only represents factors that emerged from the group of stakeholders involved in the group modeling process and may have omitted other important factors for determining the specified outcomes (e.g., the role of food systems). Moreover, the contribution of a school-based meal program and nutritional practices may not entirely account for the specified long-term outcomes, given the multifactorial causal factors that contribute to NCD incidence and the individual variations in how these factors combine to lead to NCDs.

## CONCLUSION

Adoption of SNPs in Uzbekistan and Kyrgyzstan is a first and important step to addressing nutritionally related NCD risk factors among school-aged children. However, both countries have experienced multiple challenges in the large-scale implementation of SNPs. Through a multimethod approach, we described TOCs for SNPs in Uzbekistan and Kyrgyzstan to identify key interventions, outcomes, implementation gaps, and assumptions that should be addressed and targeted with implementation strategies to facilitate the large-scale implementation effectiveness of these policies in both countries and other countries in the Central Asian region. Beyond SNPs, there is scope for broader and more comprehensive nutrition reforms that will entail the whole of government and sectors to address the nutrition needs of specific populations and both the proximal and distal causes of nutritionally related NCDs, including causes in the food systems and environment.

## Notes

**Peer Reviewed**

**First Published Online:** July 11, 2024.
